# “Bayis Ilh Tus – a strong breath” a community-based research project to estimate the prevalence of chronic obstructive pulmonary disease in remote and rural first nations communities in Canada: research protocol

**DOI:** 10.1186/s12939-020-01240-1

**Published:** 2020-07-24

**Authors:** Justin Turner, Travis Holyk, Karen Bartlett, Benna Rathburn, Barbara Karlen, Francis Ervin, Jennifer Wilson, Pat G. Camp

**Affiliations:** 1 Rehabilitation Sciences Graduate Program, University of British Columbia, Vancouver, Canada; 2 Centre for Heart Lung Innovation, University of British Columbia, St. Paul’s Hospital, 166-1081 Burrard Street, Vancouver, V6Z 1Y6 Canada; 3 Carrier Sekani Family Services, Prince George, Canada; 4 School of Population and Public Health, University of British Columbia, Vancouver, Canada; 5 Ridge Meadows Hospital, Fraser Health Authority, Maple Ridge, Canada; 6 Fraser Health Authority, Surrey, Canada; 7 Department of Physical Therapy, University of British Columbia, Vancouver, Canada

**Keywords:** Chronic obstructive pulmonary disease, Prevalence, First nations

## Abstract

**Background:**

Respiratory health conditions appear to be more common among First Nations people versus non-First Nations people in Canada. However, the prevalence of chronic obstructive pulmonary disease (COPD) and its associated risk factors in First Nations communities are unknown. This project aims to estimate the prevalence of COPD in several First Nations communities in British Columbia, Canada and to characterize respiratory symptoms, COPD risk factors, and healthcare utilization.

**Methods:**

This project is approved by both the University of British Columbia and Carrier Sekani Family Services Research Ethics Boards. We will randomly sample 220 adults, 30 years and older, from 11 participating First Nations. Each participant will complete pre- and post-bronchodilator spirometry tests and the adapted American Thoracic Society Epidemiological Questionnaire with items about smoking history, respiratory symptoms, co-morbidities, and exposures, in order to identify the presence of COPD and its associated individual, occupational, and community risk factors. Homes will be assessed for air quality measures including particulate matter, carbon monoxide and carbon dioxide, and humidity. Health care utilization will be abstracted from the electronic medical record.

**Discussion:**

This is the first project in Canada to estimate the prevalence of COPD in First Nations communities using a random-sampling approach to recruitment. Additionally, although this study will collect detailed information on smoking history, we will also characterize past and current risk factors beyond cigarette smoking. Finally, our methodology ensures that the benefits to the communities are realized during the study period. Individual results will be shared with individuals and health providers to facilitate care. Air quality results will be sent to each Nation’s governing council to facilitate remediation where necessary.

**Trial registration:**

The study has been retrospectively registered at clinicaltrials.gov (NCT04105088).

## Background

For the First Nations people in Canada, many determinants of disease, including chronic lung disease, are fundamentally related to the ongoing impacts of colonialism and, more specifically, government practices including forced attendance at residential schools and removal of children from their families for placement in to non-Indigenous adoptive homes [1]. With respect to chronic obstructive pulmonary disease (COPD), although the First Nations people have a wealth of knowledge related to health and wellness [2], the impacts of colonization and subsequent past and current harmful government policies introduced several individual- and population-level factors which negatively influence lung health and increase the risk for COPD.

Cigarette smoking (also termed non-traditional tobacco smoking in First Nations settings) is the most common individual lifestyle factor known to cause COPD [3] and was introduced to many communities post-colonization. Across Canada, more First Nations people smoke cigarettes compared to the general population (39% versus 20.5%) [4]. Moreover, the rate of cigarette smoking among First Nations has not substantially declined from 1997 to 2018 [5], while the smoking prevalence among all Canadians has decreased significantly across the same time period [4]. In the Canadian province of British Columbia, the current prevalence of cigarette smoking in Indigenous populations is approximately twice that of non-Indigenous populations (39–40% versus 18–23%, respectively) [4] with a similar trend seen for marijuana smoking [6], further increasing their risk.

Several population-level factors also negatively impact First Nations people’s lung health. Children who attended residential schools were exposed to poor air quality in unheated, poorly-ventilated buildings, had minimal access to appropriate nutrition or health care, and subsequently had high rates of childhood respiratory tract infections [2], a known risk factor for COPD [3]. As the last residential school closed in 1996, many of those children are adults today. The creation of the reserve system (where First Nations people lost their traditional territory and were forced to live in small communities) also changed seasonal patterns of hunting and harvesting and caused a reliance on western-based sources of food. This resulted in a more sedentary lifestyle, another risk factor for poor health [7].

Disparities in access to primary health care services may also influence the development of COPD in First Nations people. Limited access may be due to lack of services, especially in remote and rural communities, but are also due to decades of systemic discrimination against First Nations people [8]. Lack of appropriate primary health care can result in high emergency and acute care utilization, and indeed, the hospitalization rate for respiratory infections and asthma is at least three times higher for rural and urban First Nations than non-First Nations populations in Canada based on the 2006 Canadian Census [9], even after correcting for income level. Similarly, respiratory-related emergency use for asthma and COPD for First Nations is more than double compared to the non-First Nations population [10]. Housing problems also remain a factor, as many houses on First Nations reserves are currently in need of major repair [6], especially repair related to ventilation problems. This results in chronic exposure to mould and other pollutants which may exacerbate lung disease. Studies of indoor air quality in First Nations homes have shown high values of self-reported [11] and measured [12–14] inhaled air pollutants. Other cultural and environmental conditions exist, such as the fact that many First Nations communities are in areas where there is extensive exposure to environmental pollutants. Northern British Columbia burns more wood per household than anywhere else in the province [14] and has a high prevalence of forest fires [15] both of which contribute to many days per year with high levels of fine particulate matter (PM). Increased exposure to wood smoke and PM is associated with increased respiratory symptoms, use of medications, and health care resources [16]. Additionally, some cultural practices, such as smoking fish and meat, or indoor fires during cultural ceremonies, may increase exposure, albeit for short periods, to inhaled particles.

Despite the presence of multiple individual and community risk factors, there is little information on the prevalence and characteristics of COPD in BC First Nations. The current estimates of COPD prevalence in First Nations communities are based on physician billing records or self-reports of a physician diagnosis. A recent Alberta study found that prevalence estimates of COPD based on billing data were two times higher in First Nations people compared to non-First Nations people [17]. The 2012 First Nations Regional Health Survey stated that ~ 7% of respondents reported they had physician-diagnosed chronic bronchitis [6]. However, the “chronic bronchitis” diagnosis is not a proxy for COPD, as it is a symptom-based diagnosis without measurement of lung function. It is well known that physician billing data or self-reports are not accurate estimates of prevalence -- in the non-Indigenous population, the true prevalence of COPD is approximately three times higher than estimates based on self-report or health administrative databases [18–20]. The Canadian Health Measures Survey found that ~ 70% of individuals with mild to moderate COPD in the overall population have not been diagnosed despite many of those individuals being symptomatic [21].

The higher smoking prevalence in First Nations communities, combined with the cumulative impacts of colonization suggest that the prevalence of COPD in these communities is at least as high as that seen in non-Indigenous communities (which is estimated at 12% of adults 30+ years old) [19]. Without an estimate of the prevalence of COPD in the population, First Nations health services are currently limited in their ability to provide appropriate COPD primary care and chronic disease management services to their members. It would also be useful to understand the current exposure to respiratory risk factors in order to develop strategies to reduce the risks of exposure. Therefore, the purpose of this project is to estimate the prevalence of COPD and characterize current risk factors in several First Nations communities in the province of British Columbia, Canada.

## Methods

The research team is a longstanding partnership between researchers at the University of British Columbia (UBC), the participating First Nations, and Carrier Sekani Family Services (CSFS) – the latter being an organization that provides direct health and social services to First Nations communities in North-Central BC. Community involvement was achieved as the study concept and design was reviewed, revised, and approved by the CSFS Community Advisory Board which is made up of a representative member of the public from each participating First Nation. Research projects undertaken with CSFS require this approval from and engagement with the CSFS Community Advisory Board, which ensures study processes are supported by CSFS, data is owned by the community, and all research provides tangible benefits to the First Nations communities. This decolonizing methodology [22] is mandated in Chapter 9 of the *Tri-Council Policy Statement*, titled “Research Involving the First Nations, Inuit and Métis Peoples of Canada” [23], and forms part of our approved ethics review for this study.

This study will recruit adults 30 years and older from 11 First Nations communities in remote and rural locations in northern British Columbia. The study will consist of detailed questionnaires, lung function measurement, air quality sampling, building assessments, and community-engagement activities. Each eligible adult within a randomly-selected household will be invited to participate and provide informed consent. Data collection will occur between September to April in the 2017–2023 study period in order to obtain air quality measures during high occupancy periods and when indoor heating is likely to occur.

### Sample size and household/individual sampling

We used the Statistics Canada 2016 Census [24], the First Nations Profile [25] and the CSFS community registry to determine our estimated target population to be 1600 adults age 30+ years living in the 11 First Nations. Assuming the prevalence is 13%, a sample size of 220 adults from a population of ~ 1600 would result in a 95% confidence interval of prevalence with a half width no more than 0.05 if the intra-cluster correlation is ≤0.25. This estimate takes into account the fact that household residents are likely to be related meaning that each participant is not independent. On average, there are 2–3 adults per First Nations household [6], therefore approximately 88 households will be needed. We have planned for an additional 20% more households to ensure enough participants.

We will recruit a specific number of people from each community, which will be proportional to the adult population contribution of that community to all the CSFS communities. We estimate that we will be able to recruit 1–2 adults per household. Using a random-number generator, each household is then randomly placed in numbered order. Starting at the top of the list, we then approach the adults in that household with an invitation to participate. If that household declines, we move to the next household on the list. Based on previous Canadian COPD prevalence studies using random population sampling and direct measures of lung function [3, 18, 19], the prevalence of COPD in Canada is 8.1% among all adults, and 15% among ever-smokers. Due to the high prevalence of smoking in First Nations communities, the true prevalence of COPD is likely greater than 8%, so we have used 13% as our estimate for sample size calculations.

### Spirometry measurements

Lung function data will be collected in the seated position using a Koko Legend spirometer (CareFusion, Toronto, Canada) before and after administration of 400 μg of the inhaled bronchodilator salbutamol via a metered-dose inhaler with a spacer, following American Thoracic Society (ATS) standards [26]. Each measure will be reviewed by a board-certified respirologist (co-authors FE & JW) who will be blinded to details of medical history and risk factors. The presence of a ratio of post-bronchodilator forced expiratory volume in the first second (FEV_1_) to forced vital capacity (FVC) < 0.70 will be used to determine airflow obstruction consistent with COPD. The severity of COPD will be categorized according to the GOLD guidelines [3]. There are no validated prediction equations for First Nations populations, therefore we will use Canadian prediction equations of lung function [27] which are similar to the widely-used equations in the U.S. National Health and Nutrition Examination Survey [28]. The respirologist will also make note of any other lung function abnormalities (e.g., evidence of asthma or interstitial lung disease) that require follow-up by the family physician.

### Lung health questionnaires

Each participant will complete the American Thoracic Society Epidemiological Respiratory Questionnaire [29] with additional items from the Saskatchewan First Nations Lung Health Study [11] questionnaire as well as items related to relevant occupational, environmental, and cultural exposures. These extensive questionnaires contain detailed questions related to smoking history (cigarette, marijuana, vaping, and other smoked products), childhood and family history of lung disease, respiratory symptoms, comorbid conditions, other inhaled risk factors, residential air quality, outdoor air quality,, medication use, health care utilization (HCU), activity limitation, occupational history and exposures, time spent in household and community buildings, financial security, community strengths, residential school attendance and self-report of discrimination. The additional questions were developed in consultation with the Community Research Advisory Committee, and include Indigenous perspectives in maintaining one’s own health by asking: What do you do to keep your lungs healthy? If you have problems with your breathing, what do you do to get better? What are the strengths of your community?

### Previous physician diagnosis of COPD

CSFS provides primary health services to all residents living on reserve. Care provided by family physicians, nurse practitioners, nurses and other health care professionals is detailed in the CSFS electronic health record (EHR). Participants who allow access of their EHR will be categorized as having physician-diagnosed COPD if any of the following occur in their medical records: 1) COPD, chronic obstructive lung disease, chronic airflow limitation, chronic bronchitis, or emphysema in the diagnosis fields; 2) history of an acute exacerbation of COPD (AECOPD) with or without hospitalization; or 3) six or more consecutive months of the COPD-specific long-acting bronchodilator tiotropium bromide prescribed without a diagnosis of asthma and 10 or more pack years of cigarette smoking.

### Health care utilization

Using the EHR system, we will count the number of respiratory-related visits in the previous 2 years to the primary care physician, nurses, and nurse practitioners. Eligible respiratory-related events will include lower respiratory tract infections, bronchitis, shortness of breath, and productive cough. We will also ask participants how many times they visited any Emergency Department and/or were admitted to any hospital for respiratory-related problems in the previous year.

### Household and community measures of air quality

We will measure air quality in occupied residences and community buildings (including health centres, Band offices, and community centres). The major sources of indoor air pollution are combustion products (second-hand smoke, wood burning) and pollutants related to excess moisture in interior structures and furnishings. We will collect data via direct observations and physical samples. Participants also consent to the home inspection visits and sampling of household indoor air. We will get permission to sample community buildings from Chief and Council. We will also meet with Chief and Council to determine the appropriate manner of reporting air quality findings back to each community.

We will use checklists comprised of items from the United States General Services Administration Checklist for the Routine Inspection of Buildings [30] and co-author Bartlett’s previous bioaerosol work [31] to assess the presence and use of exhaust vents, signs of uncontrolled moisture, occupancy levels, and presence/use of indoor combustion appliances in residential and community buildings. Air and dust samples will be taken at the same time the building questionnaires are completed. Real time measurements will be taken of carbon monoxide (CO), carbon dioxide (CO_2_), temperature, and relative humidity using a Q-Trak monitor (TSI Incorporated, Shoreview, Minnesota, USA). The concentration of CO_2_ relative to the number of occupants can be used to calculate air exchange rate. CO, on the other hand, is a hazardous gas which is produced during incomplete combustion of carbon-based fuels. It can accumulate in residences with low gas exchange. Relative humidity can be predictive of conditions which would allow fungi to grow on interior building materials. Respirable PM < 5.0 μm will be measured using a handheld laser particle counter (bin sizes 0.3, 0.5, 1.0 and 5.0 μm) (Kanomax, Andover, New Jersey, U.S.A). Settled dust samples will be collected to assess the presence and amount of inhalable bioactive molecules including antigens and endotoxin. We will collect a 15 ml dust sample from each of three rooms in the house (typically a bedroom, the living room, and an additional room (or commonly-used space if a community building) using a portable vacuum with a sampling sock (pore size 5-10 μm) [32]. Each dust sample will be sealed in a separate Ziplock bag, returned to the lab, and sieved to remove material > 300 μm, with the resulting dust extracted (100 mg/2 ml 0.05% Tween 20 in pyrogen-free phosphate buffered saline pH 7.4) overnight and the filtered extract stored at − 20 °C until analysis.

A standard unit of extracted dust will be analyzed using enzyme-linked immunosorbent assay (ELISA) reagents obtained from Indoor Biotechnologies (https://inbio.com) and read on a Molecular Devices SpectraMax 190 microplate reader. The following compounds will be examined from the same house dust sample: antigens (housedust mite Der p1 and Der f1; cat Fel d1; dog Can f1; mouse Mus m1) and mould antigens (*Alternaria alternata* Alt a1; *Aspergillus fumigatus* Asp f1). The limit of detection of ELISA antigen determination is 0.4 ng/mL extract. The pro-inflammatory molecule, endotoxin, will be quantified using a kinetic, chromogenic reaction using Limulus Amoebocyte Lysate (LAL) reagent (Kinetic QCL, Lonza Walkersville Inc.). Samples will be also stored as baseline measures for remediation studies.

### Data analysis

Using pooled data, descriptive statistics will be used to characterize participants and their communities, including lung function, symptoms, HCU and risk factors. The prevalence of COPD will be calculated as the ratio of COPD cases, based on lung function, to the sampled population. To get a representative estimate of the population prevalence of COPD in remote and rural First Nations communities in BC, we will consider the cluster design and sampling weight, which is the inverse of the likelihood of being sampled. The sampling weights in this study will be computed as the ratio of household size to total population in each community. These weights will be included in the calculation of prevalence with 95% confidence interval estimation. Under-diagnosis of COPD will be estimated by confirming the prevalence of COPD based on spirometry with the medical diagnosis in the EHR.

Secondary analyses will estimate the levels of indoor air pollutants and compare them to consensus guidelines established by the American Society of Heating, Refrigeration and Air-Conditioning Engineers [33, 34] and Health Canada [35]. We will characterize the levels of inhaled pollutants from individual, residential, occupational, cultural/community sources as well as respiratory symptoms, airflow obstruction, and respiratory-related HCU.

### Ethics and dissemination

This study has been approved by both the UBC and CSFS Research Ethics Boards and the study will follow Indigenous research principles, as described by the CSFS guide for researchers [36]. These principles are in line with the “Tri-Council Policy Statement on Ethical Conduct of Research Involving Aboriginal Peoples” [23] and the First Nations Information Governance Centre’s “First Nations Principles of OCAP (Ownership/Control/Access/Possession)” [37]. In addition, the CSFS Community Research Advisory Committee has approved the project and agreed to guide the research.

We will develop an integrated knowledge translation (KT) strategy that reflects our different audiences. Ongoing project updates will be shared with the communities via their Facebook pages and the CSFS website (www.csfs.org), newsletter, and Twitter account (@CarrierSekaniFS). The study results will be communicated through peer-reviewed publications, bulletins, and presentations to the First Nation, health care and research communities. If the prevalence and underdiagnosis of COPD is high, sharing these results with the health authorities will increase the awareness of COPD as an urgent health need. The individual lung function results will be shared with participants, and we will ask their permission to share results with the health care centres for further follow-up. We will consult with our partner Chiefs and Council regarding the air quality results. We will also create educational posters and visit each community to share the results and plan for next steps. Finally, we will create a video that highlights the project steps and summarizes results using meaningful stories of project participants.

## Discussion

This paper describes the methods for an epidemiological research study, made possible via a partnership between UBC, CSFS, and the participating member Nations, and conducted in a culturally safe way. Indigenous research principles, as determined by CSFS and their member Nations, state that all research must address the goals and needs of the communities, and provide tangible benefits to the communities and to CSFS. We have ensured that this project meets those expectations by first raising awareness of the issues via lung health events, obtaining permission and guidance through community advisory boards, ensuring the ethical conduct of the study based on both Indigenous and western-based ethical guidelines, having all research team members participate in CSFS cultural safety training, and keeping our obligations to the community in terms of ensuring benefit through spirometry directly within remote communities that do not have access to such a service without significant travel. We also reinforce that although this research is focused on identifying the burden of COPD within the community, we work to avoid a ‘deficit’ lens – i.e. the misguided viewpoint that the First Nations communities have low agency and are in need of ‘fixing’ by external sources. This study is viewed by the communities and the research team as an opportunity to gain knowledge to improve lung health, in order to support future planning for health care management, as determined by the community and CSFS.

There are many strengths to this project. First, to our knowledge this is the first project in Canada to estimate the prevalence of COPD in First Nations communities using a random-sampling approach to recruitment. Although more labour- and time-intensive, it is the optimal method to ensure the most accurate estimates. Second, we ensure quality control for the spirometry measurements by having: 1) pre- and post-bronchodilator measurements to reduce the risk of errors in categorization; 2) a board-certified respirologist interpreting the results; and 3) frequent quality control assessments throughout the study period. Another strength of this project is that although we collect detailed information on smoking history, we also characterize past and current risk factors beyond cigarette smoking, including childhood exposures, occupational factors, household and outdoor exposures, and the impact of discrimination and access to health services. Finally, our study ensures that the benefits to the community are realized during the study period – we offer lung testing to all adults who are interested; the test results become part of the health record; all lung function abnormalities (COPD and otherwise) are followed up by the primary care team; and spirometers and training are provided to each community for their ongoing use.

In conclusion, this study will provide an estimate of the burden of COPD, including prevalence, and under-diagnosis. We will also characterize risk factors, and respiratory morbidity in First Nations communities in Northern British Columbia. The results from this study will increase the awareness of COPD, and will support health service delivery planning and prevention efforts in the communities. Other First Nations communities in Canada may also find the protocol and subsequent results helpful as they develop chronic disease management strategies for COPD in their communities.

## Data Availability

Not applicable.

## Abbreviations

AECOPD: Acute exacerbation of chronic obstructive pulmonary disease
ATS: American Thoracic Society
BC: British Columbia
COPD: Chronic obstructive pulmonary disease
CSFS: Carrier Sekani Family Services
EHR: Electronic health record
ELISA: Enzyme-linked immunosorbent assay
FEV: Forced expiratory volume
FVC: Forced vital capacity
GOLD: Global Initiative for Chronic Obstructive Lung Disease
HCU: Health care utilization
PM: Particulate matter
UBC: University of British Columbia
